# The Influence of Dental Occlusion on Dynamic Balance and Muscular Tone

**DOI:** 10.3389/fphys.2019.01626

**Published:** 2020-01-31

**Authors:** Sonia Julià-Sánchez, Jesús Álvarez-Herms, Rafel Cirer-Sastre, Francisco Corbi, Martin Burtscher

**Affiliations:** ^1^Faculty of Physical Activity and Sport Sciences, European University, Madrid, Spain; ^2^Institut Nacional d’Educació Física de Catalunya, Universitat de Lleida, Lleida, Spain; ^3^Department of Sport Science, University of Innsbruck, Innsbruck, Austria

**Keywords:** dental occlusion, dental occlusion balanced, dynamic balance, postural control, muscle properties, Myoton

## Abstract

Excellent postural control is essential to improve the physical performance of athletes. Stability of the body during motor tasks depends on different physiological systems. The influence of dental occlusion on body balance has been widely investigated in the past few years. It has been suggested that this relationship is strengthened by disturbing environments for balance control (i.e., unstable platform, fatigue, development tasks.). Moreover, dental occlusion may influence the muscle tone of both masticatory and postural muscles, which are involved in the preservation of balance. Therefore, we attempted to determine whether (i) there are differences in dynamic balance assessed by the modified star excursion balance test between opposed dental occlusion conditions (dental contact: intercuspal position/no dental contact: cotton rolls mandibular position) and (ii) dental occlusion influences the biomechanical and viscoelastic properties of the masticatory and postural muscles assessed with MyotonPRO^®^. Thirty physically active subjects were recruited for the study. The main findings were the following: (i) the Star Excursion Balance Test composite score was significantly higher for measurements made in cotton rolls mandibular position (*p* < 0.001) and also in subjects showing a correct occlusion (*p* = 0.04), and (ii) the biomechanic and viscolelastic properties of selected muscles showed different trend according to the presence of malocclusal traits. It is concluded that dental occlusion conditioned both dynamic stability and the biomechanic and viscoelastic properties of the analyzed muscles.

## Introduction

Postural control is a coordinative ability influencing the efficient competence of motor tasks in many essential daily activities from children to elderly (Horak, 2006; Stodden et al., 2008). It has been defined as the act of maintaining, achieving, or restoring a state of balance during any posture or activity (Pollock et al., 2000) and depends on different complex skills based on the interaction of dynamic sensorimotor processes (i.e., visual, vestibular, and proprioceptive systems) (Fitzpatrick and McCloskey, 1994). Postural stability can be maintained in balanced equilibrium by passive resting muscular tone that derives from intrinsic molecular viscoelastic properties (Masi and Hannon, 2008). These passive viscoelastic muscle properties are independent from central nervous system (CNS) activation and are conditioned by mechanical myofascial tissue properties (Mense and Masi, 2010; Nair et al., 2016). Those muscle properties contribute importantly to maintain postural stability in balanced equilibrium position, such as the muscle stiffness contributes in the maintenance of body balance during quiet standing (Winter et al., 2001). In addition, on an unstable ground, the muscle tone and elasticity properties correlate positively with sway characteristics (Vain et al., 2015). Within the physical competence, the coordination of movements to stabilize the center of body mass during self-initiated and externally triggered disturbances of stability is critically important (Hrysomallis, 2011). Several factors as central/peripheral fatigue (Paillard, 2012), muscle strength and proprioception (Sanchez-Ramirez et al., 2013), biorhythms (Gribble et al., 2007), the visual–vestibular interaction (Dichgans and Brandt, 1978), age (Alexander, 1994), foot postures (Cote et al., 2005), cortical control (Jacobs and Horak, 2007), neuroendocrine responses (Ma et al., 1992), and the stomatognathic system (Julià-Sánchez et al., 2019) are strongly involved in the maintenance of postural control. The effectiveness of the individual body balance ability will finally depend on both the complexity of the postural tasks developed and the capability of the subject’s postural control system, which involves many of the underlying physiological systems mentioned above.

Literature regarding the relationship between the stomatognathic system and the body posture has grown at a huge rate in the past few years. However, conflicting data have been reported, and results of recent reviews have benchmarked for (Cuccia and Caradonna, 2009; Moon and Lee, 2011) and against (Michelotti et al., 2011; Manfredini et al., 2012; Perinetti et al., 2013) this clinical correlation. Recently, it has been argued that dental occlusion may differentially contribute to the postural control depending on the external disturbances, with a greater contribution to the postural control when more difficult conditions are present (i.e., unstable conditions and muscle fatigue) (Tardieu et al., 2009; Julià-Sánchez et al., 2015, 2016, 2019). Interestingly, it has recently been proposed that the stomatognathic system may also contribute to the postural balance in ∼2% (Solovykh et al., 2012). This contribution might, *a priori*, be insignificant for the physical competence of the general population. However, it may be of great importance in sport performance, contributing to the difference between winners and losers in sport where the body balance is determinant. Shooting, combat disciplines, gymnastics, or skiing are several examples of sports where the postural control is a basic feature to reach an optimal competitive physical performance. On the other hand, a poor balance control has been related to promote higher risk from suffering injuries during exercise (Hrysomallis, 2007). A plausible explanation for the influence of dental occlusion on the maintenance of posture is the muscle interconnection between the masticatory and postural muscles. In fact, the influence of dental occlusion on the muscular activity has been the subject of numerous studies from early publications of Hickey et al. in the 1950s measuring electromyographically the muscles controlling mandibular movements with different occlusal schemes (Hickey et al., 1959). Moreover, evidence to date suggests strong interrelatedness of jaw, neck, and trunk muscle activity (Davies, 1979; Ehrlich et al., 1999; Giannakopoulos et al., 2013a) and seems that neck muscles contract rhythmically with the mandibular muscles during various activities of the mandible (Davies, 1979). The co-contraction behavior of the masticatory and neck and trunk muscles has been reported under jaw clenching and tooth grinding (Davies, 1979; Pirttiniemi et al., 1989; Clark et al., 1993; Ehrlich et al., 1999; Rodríguez et al., 2011; Giannakopoulos et al., 2013a) and also during submaximum bite forces (Hellmann et al., 2012; Giannakopoulos et al., 2013b). Interestingly, the interconnection between the neck and masticatory muscles may result from complex neurophysiological interactions rather than a simple biomechanical muscle chain coupling (Giannakopoulos et al., 2018). The functional coupling between dental occlusion and the masticatory and neck muscles has been subject of extensive study. A recent review concludes that occlusal features can affect the electrical signal recordings of masticatory muscles (Trovato et al., 2009). Thus, altered dental occlusion may influence the muscle activity of masticatory muscles (i.e., masseter and temporalis) (Ferrario et al., 2000), but also experimental occlusal discrepancies influence on both masticatory (Baba et al., 1996) and neck (SMC) muscle activity (Ferrario et al., 2003). Alterations in the quantity and quality of fibers affecting the structure and biomechanical properties of neck and back myofascial tissues could change optimum levels of musculotendinous stiffness that are highly correlated with muscle performance (Wilson et al., 1994) and greatest level of injuries (Zinder and Padua, 2011). Recently, Ivanov et al. (2018) observed variations in the electromyography activity of temporalis and masseter muscles during orthodontic treatment that could influence postural balance system.

Because dental occlusion seems to strongly influence muscle tone and postural balance, the analysis of the characteristics of masticatory and postural muscles in patients with different malocclusal traits would aid to know the specific effect of the occlusal traits on the muscle characteristics and posture. We hypothesized that subjects with correct occlusion (without dental malocclusal traits) will have appropriate levels of musculotendinous stiffness and viscoelastic properties and thus better balance control than subjects with dental malocclusion (likely showing higher stiffness levels in the ES). Therefore, this study aimed (i) to determine if there are differences in dynamic balance measured with the modified star excursion balance test (MSEBT) between opposed dental occlusion conditions [dental contact: intercuspal position (ICP)/no dental contact: cotton rolls (CR) mandibular position] and (ii) to analyze whether dental occlusion influences in the biomechanical properties of several masticatory and postural muscles.

## Materials and Methods

### Participants

A convenience sample of 30 physically active subjects (19 male, 11 female; 22.9 ± 3.7 years; height, 1.72 ± 0.11 m; body mass, 67.4 ± 10 kg; body mass index, 22.62 ± 1.65) was recruited for the study. Study participants were recruited according to the following inclusion criteria: being sport science students of INEFC Lleida and amateur athletes, training on 3 days/week plus eventual competition. Exclusion criteria were the following: history of musculoskeletal problems or any vestibular impairment, taking regular medication, smoking or drinking alcohol, or self-reported signs and symptoms of temporomandibular disorders (subsequently proved in the occlusal analysis). Table 1 shows the occlusal characteristics of each participant.

**TABLE 1:** Occlusal traits of the subjects (ORT, orthodontic treatment; MLD, midline deviation; CRW, crowding; DIA, diastema; MT, missing teeth; ANGLE, Angle Class; OPB, open bite; OVB, overbite; OVJ, overjet).

Subject ID	ORT	MLD	CRW	DIA	MT	ANGLE	OPB	OVB	OVJ	VER
1	–	–	+	–	–	III	–	+	+	+
2	+	–	+	–	–	III	+	+	+	+
3	+	–	–	–	–	I	–	–	–	–
4	+	–	–	–	–	I	–	–	–	–
5	+	–	–	–	–	I	–	–	–	–
6	+	–	–	–	–	I	–	–	–	–
7	+	–	–	–	–	III	–	+	–	–
8	+	–	+	+	+	II	–	+	–	–
9	+	–	–	–	–	I	–	–	–	–
10	–	+	–	–	–	III	–	–	+	–
11	–	+	+	–	–	III	–	+	+	+
12	+	–	–	–	–	III	–	–	+	–
13	+	+	–	–	–	III	+	–	+	–
14	–	+	+	–	–	III	–	+	+	+
15	+	–	–	–	–	III	–	–	+	–
16	+	+	+	–	–	III	–	–	+	+
17	+	+	+	+	+	III	+	–	+	–
18	+	–	–	–	–	III	–	–	+	–
19	+	+	–	+	–	I	–	+	+	–
20	+	+	–	–	–	III	–	+	+	–
21	–	+	–	–	–	III	–	+	+	–
22	+	+	–	–	–	I	–	–	–	–
23	–	–	+	–	–	III	+	+	+	–
24	–	+	–	–	–	III	–	–	–	–
25	+	+	–	–	–	III	–	–	–	–
26	+	+	–	–	+	III	+	–	+	+
27	–	–	–	+	–	I	–	–	–	–
28	–	+	+	–	–	I	–	+	+	+
29	+	–	+	–	–	III	+	–	+	–
30	–	+	–	–	–	I	–	–	–	–

Subjects were advised to avoid excitatory substances (i.e., coffee, tea) and physical activity for 72 h before the test. All the participants gave their written informed consent according to the updated Declaration of Helsinki, and the project protocol was approved by the university’s ethics review board.

### Experimental Design

#### Testing Apparatus

A hand-held myotonometer MyotonPRO^®^ (Myoton Ltd., Estonia) was used to measure the muscle tone, the biomechanical (stiffness and elasticity), and the viscoelastic (mechanical stress relaxation time) properties of the following muscles: masseter (M), sternocleidomastoid (SCM), and erector spinalis longissimus (ES).

The MyotonPRO^®^ measures the viscoelastic response of the muscle due to a brief (15 ms) mechanical impulse (force, 0.4 N) on the skin surface above the muscle. The mechanical deformation is delivered by the device testing end (*d* = 3 mm) held perpendicular to the skin surface (Treffel et al., 2016). The device was used in multiscan mode, where one measurement corresponded to the mean of three mechanical taps (Pruyn et al., 2016). The validity and reliability of the Myoton measures in limb, trunk, and orofacial musculature are well documented (Treffel et al., 2016; Lo et al., 2017).

We investigated four parameters computed in real time by MyotonPRO^®^ software: oscillation frequency (the muscle tone or the mechanical tension in a relaxed muscle), dynamic stiffness (the resistance of the muscle to contraction or an external force that changes muscle’s initial shape), and logarithmic decrement (muscle elasticity in terms of the recovery of the muscle’s initial shape after contraction or removal of an external force) (biomechanical properties), and mechanical stress relaxation time (the time for the muscle to restore its initial shape after external force is removed (viscoelastic properties) (Schneider et al., 2015; Ko et al., 2018).

For the assessment of body stability, MSEBT was used. This test was adapted from SEBT, a functional screening tool widely used for the assessment of dynamic stability and also for the detection of functional performance deficits associated with lower extremity pathologies (Hertel et al., 2000). Although the original SEBT consists of eight directions, conventional testing procedures have adopted a modified version of the test, using the anterior, posteromedial, and posterolateral directions (Plisky et al., 2006) due to its high level of representativeness (Hertel et al., 2006). The MSEBT version has been widely used in sports as injury-prevention screening tool (Gkrilias et al., 2018) and to evaluate dynamic balance (Hemmati et al., 2017; Denehey et al., 2018).

#### Testing Procedure

**FIGURE 1:**
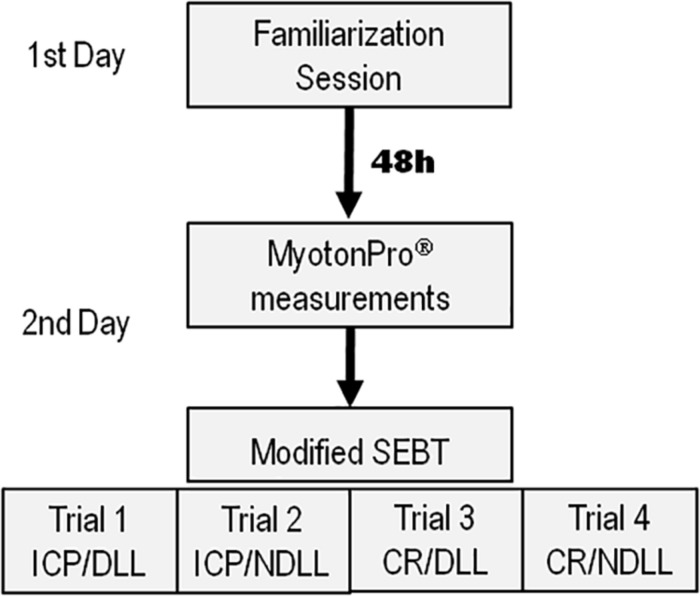
Testing procedure scheme for the 2 days of the study. 1st day (familiarization session), 2nd day (experimental session). For each trial, subjects were randomly assigned to the dental occlusal condition: CR (Cotton Rolls); ICP (Intercuspal Position).

Subjects reported to the laboratory on 2 days separated by 48 h. The first day was a familiarization session, while the second was the experimental session (Figure 1). All measurements were obtained at a controlled temperature of 22°C and 45–55% humidity and were all done in the morning to avoid the influence of biorhythms on dynamic balance, which has been seen to be better in the morning (Gribble et al., 2007).

The occlusal analysis was conducted on the familiarization session, as well as the anthropometric measurements, including the subject’s limb length. This measurement was recorded with subjects lying supine, from the most distal end of the anterior superior iliac spine to the most distal end of the lateral malleolus on each limb (Gribble and Hertel, 2003). During the familiarization session, the subjects were instructed in the procedure of execution of the MSEBT with verbal and visual demonstration. Participants were scheduled for the date and time of the testing session.

On the experimental session, the muscle tone and biomechanical properties of selected muscles were recorded within a non-invasive approach with the MyotonPRO^®^. During the measurements, the subject stood in an upright posture to measure the muscle properties influenced by posture (Pruyn et al., 2016). Subjects were placed on a hard, stable, and smooth surface in accordance with the Association Française de Posturologie standards (Bizzo et al., 1985). All measurements were recorded with subjects barefoot, in a Romberg position (standing upright with the hands to the sides of the body), and looking at a fixed point drawn on the wall at their eyes height, at an approximate distance of 90 cm (Lê and Kapoula, 2008). The feet were placed in slight abduction (30°), and the head was kept in a neutral position (Simoneau et al., 1992).

Anatomic landmarks were marked with a pen (bilaterally on the right and left muscles) to assess that all the measurements were taken on the same muscle point. Measurements were recorded: (i) masseter, perpendicular to the surface of the main portion of the masseter, 20 mm from the inferior edge of the mandibular angle (gonion); (ii) SCM, at the lower one-third of the line connecting sternal notch and mastoid process; and (iii) ES, two finger width lateral from the L1 spinous process. Anatomic locations were marked on relaxed muscles selected according Surface Electromyography for the Non-invasive Assessment of Muscles guidelines (Hermens et al., 2000). All collected values were downloaded to a computer for data analysis.

After that, subjects completed a MSEBT, and measurements were taken by the same experienced rater. The tests were carried out for two dental occlusion conditions: (i) dental contact, setting dental occlusion in ICP by asking the subject to clench his/her teeth and (ii) without dental contact, using cotton rolls (8 mm thick) between the two arches placed from the canines to the molars, CR mandibular position. The order of the dental occlusion condition for the two testing trials was randomly assigned. During the execution of the tests, the subjects stood on one lower extremity, with the most distal aspect of their great toe at the center of the grid. The subjects were then asked to reach in the anterior, posteromedial, and posterolateral direction while maintaining their single-limb stance. The subjects underwent four practice trials on each limb for each of the three reach directions of the test (Robinson and Gribble, 2008).

Once the practice trials had finished, the experimental test began, and both dental occlusion conditions were tested for each lower limb. Subjects underwent MSEBT with the CR or the ICP condition for the dominant and non-dominant lower limb. The process was then repeated while maintaining the opposite dental occlusion condition (CR/ICP). The order of dental occlusion conditions and limb testing was counterbalance randomized by the tester. The recovery between tests for each participant was fixed at 2 min according to previous studies employing the SEBT (Robinson and Gribble, 2008). The examiner visually recorded the most distal location of the reach foot as it contacted the grid in three directions. The trial was discarded, and the subject repeated the testing trial if (i) the subject was unable to maintain single-limb stance, (ii) the heel of the stance foot did not remain in contact with the floor, or (iii) the reach foot did not return to the starting position before reaching in another direction (Filipa et al., 2010).

The SEBT composite score was calculated by dividing the sum of the maximum reach distance in the anterior (A), posteromedial (PM), and posterolateral (PL) directions by three times the limb length (LL) of the individual, then multiplied by 100 {[(A + PM + PL)/(LL × 3)] × 100} (Filipa et al., 2010).

#### Occlusal Analysis

The analysis of the oral cavity was conducted by the same experienced dentist to avoid interexaminer variability. The following occlusal traits were recorded: angle classification according to class I (neutrocclusion), class II (retrognathism), and class III (prognatism); crowding ≥ 3 mm; midline deviation; presence of space or gap between two teeth (diastema); missing teeth (excluding third molars); incomplete contact between front upper and lower teeth (open bite); extent of vertical overlap of the maxillary central incisors over the mandibular central incisors (overbite); and distance between the maxillary anterior teeth and the mandibular anterior teeth in the anterior–posterior axis (overjet ≥ 4 mm), as well as previous orthodontic treatment. For the analysis of results, we considered the influence of each individual malocclusal trait on the SEBT score and on the muscle properties studied. After that, we also grouped the subjects according to those showing correct occlusion (angle I and no malocclusal traits) and those with malocclusion (subjects with one or more malocclusal traits), and then, we considered the potential influence of having an correct occlusion or malocclusion on the SEBT score and on the muscle properties.

### Statistical Analysis

Descriptive data are presented as mean ± standard deviations (SD) unless otherwise stated. Statistical analyses were conducted in R, Version 3.5.0 (R Development Core Team) using the lme4 package to fit linear mixed-effects models by restricted maximum likelihood. Residuals were examined to satisfy the assumptions of normality and homoscedasticity. Two variables, oscillation frequency and dynamic stiffness, were log transformed to achieve a Gaussian distribution before analyses. Effect sizes were provided by calculating Cohen’s *d* with Hedge’s correction. Statistical significance was established at *p* < 0.05.

#### Model Specification

For the dynamic stability analysis, an initial model was fitted with SEBT composite score as a dependent variable, with occlusal condition (CR or ICP) and limb (NDLL or DLL) as fixed effects with random intercepts for each participant. After discarding any significant influence of limb on the SEBT composite score, limb was treated as random effects for the subsequent models. The reduced model was then fitted with each occlusal trait, the presence or absence of malocclusal traits, or the number of malocclusal traits in each subject as fixed effects, with random intercepts for participant and limb. In addition, for the myometric analysis, models were fitted with each biomechanical/viscoelastic property as a dependent variable, the presence or absence of malocclusal traits or the count of malocclusal traits as fixed effects, and random intercepts for participant and limb.

## Results

Recorded data were grouped according to (i) the association between the dental occlusion (ICP, CR, correct occlusion, and presence of malocclusal traits) and dynamic stability measured with the SEBT and (ii) the influence of dental occlusion on the biomechanical muscle properties.

### Effect of Dental Occlusion on the Dynamic Stability

**FIGURE 2:**
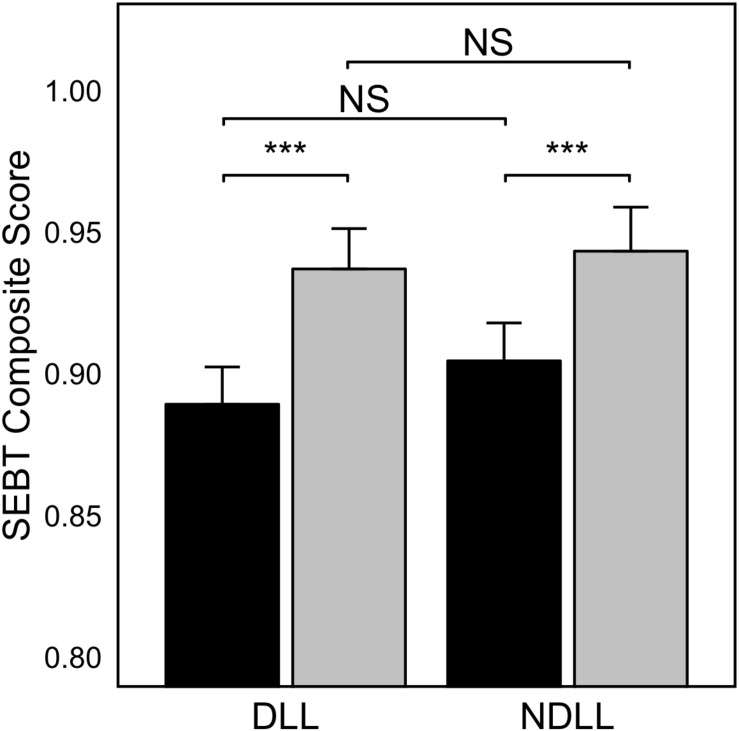
SEBT Composite score for the dominant (DLL) and non-dominant (NDLL) lower limb when comparing dental occlusion in “Cotton Rolls” mandibular position (gray bars) and Intercuspal position (black bars). Values are represented as Mean ± SE (*n* = *30*). Asterisk indicates statistically significant differences for *p* < 0.001; NS, non-significant differences.

SEBT composite score showed significant differences between measurements made in CR and ICP disregarding the dominant and non-dominant lower limb (*p* < 0.001). For each lower limb measurement, SEBT score was higher for the CR condition [NDLL, 0.90 ± 0.07 (ICP) vs. 0.94 ± 0.08 (CR), *p* < 0.001; and DLL, 0.89 ± 0.07 (ICP) vs. 0.94 ± 0.08 (CR), *p* < 0.001]. No statistically significant differences were found between the dominant and non-dominant lower limb measurements disregarding the occlusal condition [ICP, 0.90 ± 0.07 (NDLL) vs. 0.89 ± 0.07 (DLL), *p* = 0.07; and CR, 0.94 ± 0.08 (NDLL) vs. 0.94 ± 0.08 (DLL), *p* = 0.45]. Figure 2 shows an overview of the composite index measured in the dominant and non-dominant lower limb under both dental occlusion conditions.

### Occlusal Traits and Dynamic Stability

Table 2 shows an overview of the SEBT composite index when grouping the data according to the dental occlusion condition and considering the different occlusal traits of the subjects. The variable angle II occlusion was discarded, as only one subject presented this type of occlusion. Analysis of variance revealed that none of the malocclusal traits by themselves influenced the stability index measured with the SEBT. Thus, the statistically significant differences in SEBT scores can be explained by the change in the mandible position, from ICP to CR, but not for presenting any specific maloccusal trait.

**TABLE 2:** SEBT Composite Index for the two dental occlusion conditions tested (ICP, Intercuspal Position; CR, “Cotton Rolls” mandibular position) with attention to the examined occlusal traits (Mean ± SD).

	*N*	ICP	CR	*P-*value	
Angle I	10	0.92 ± 0.08	0.96 ± 0.09	<0.001	0.68
Angle III	19	0.89 ± 0.07	0.93 ± 0.08	<0.001	
Orthodontics	20	0.9 ± 0.08	0.94 ± 0.09	<0.001	0.77
No Orthodontics	10	0.89 ± 0.06	0.93 ± 0.07	<0.001	
Crowding	10	0.92 ± 0.04	0.97 ± 0.06	<0.001	0.15
No crowding	20	0.88 ± 0.08	0.93 ± 0.09	<0.001	
Midline deviation	15	0.89 ± 0.07	0.93 ± 0.08	<0.001	0.34
No midline deviation	15	0.91 ± 0.07	0.95 ± 0.09	<0.001	
Diastema	4	0.88 ± 0.05	0.94 ± 0.05	<0.001	0.57
No diastema	26	0.9 ± 0.08	0.94 ± 0.09	<0.001	
Missing teeth	3	0.88 ± 0.05	0.95 ± 0.05	<0.001	0.78
No missing teeth	27	0.9 ± 0.07	0.94 ± 0.08	<0.001	
Anterior open bite	6	0.9 ± 0.05	0.95 ± 0.04	<0.001	0.92
No anterior open bite	24	0.9 ± 0.08	0.94 ± 0.09	<0.001	
Overbite	11	0.92 ± 0.06	0.96 ± 0.06	<0.001	0.27
No overbite	19	0.89 ± 0.08	0.93 ± 0.09	<0.001	
Overjet	18	0.89 ± 0.06	0.94 ± 0.07	<0.001	0.73
No overjet	12	0.9 ± 0.09	0.94 ± 0.1	<0.001	
Version	7	0.92 ± 0.06	0.96 ± 0.07	<0.001	0.73
No version	23	0.89 ± 0.08	0.93 ± 0.08	0.007	

**N*, sample size. *P-*value indicates comparison for ICP vs. CR condition (first column) and presence vs. absence of each occlusal trait (second column).*

**FIGURE 3:**
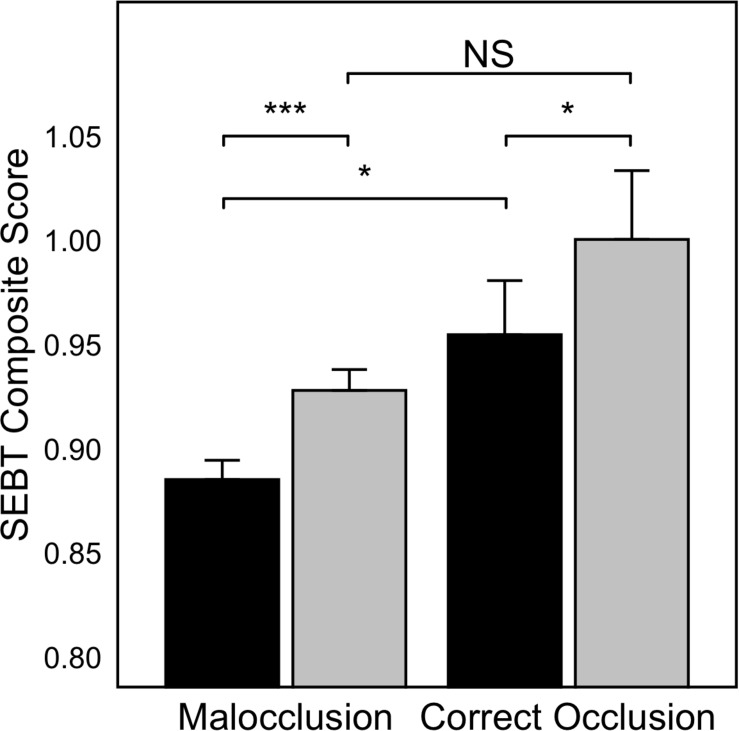
SEBT Composite score when grouping subjects in those with correct occlusion (*n* = 5) and subjects with malocclusion (one or more malocclusal traits) (*n* = 25). Values are represented as Mean ± SE. Black bars indicate ICP, gray bars indicate CR condition. *p < 0.05; ***p < 0.001; NS, non-significant differences.

The SEBT composite score when grouping the subjects in accordance with the presence (*n* = 25) or absence (*n* = 5) of malocclusal traits was significantly higher in subjects who had correct occlusion (0.98 ± 0.09) in comparison with those with malocclusion (0.91 ± 0.07) regardless of the limb measured (DLL/NDLL) and the occlusion condition (CR/ICP) (*p* = 0.04). In addition, both subgroups of participants (correct occlusion/malocclusion) improved their SEBT composite score when changing the mandible position from ICP to CR (correct occlusion: ICP, 0.96 ± 0.08; CR, 1 ± 0.1, *p* = 0.019; malocclusion: ICP, 0.89 ± 0.07; CR, 0.93 ± 0.07, *p* < 0.001). In this respect, for the measurements made in ICP condition, subjects with correct occlusion showed better balance than subjects with malocclusion (*p* = 0.047), whereas similar SEBT composite score was recorded for measurements in CR between the two groups (*p* = 0.12) (Figure 3).

### Biomechanical Properties of Muscles

Biomechanical and viscoelastic properties were different among muscles, as well as when grouping the subjects in accordance with the presence or absence of malocclusal traits. The absence of malocclusal traits, namely, correct occlusion, was positively associated with frequency in the SCM (*p* = 0.03) but negatively associated with the same property in the EC (*p* = 0.023) and M (*p* = 0.022). Stiffness was higher in the SCM of those participants with correct occlusion (*p* = 0.021) but lower in their ES (*p* = 0.019) and M (*p* = 0.020). Decrement was lower in the M of those participants with correct occlusion (*p* = 0.015) but independent of the presence/absence of malocclusal traits in the SCM (*p* = 0.487) and ES (*p* = 0.125). Relaxation, on the other hand, was higher in the ES of those participants with correct occlusion (*p* = 0.012) but lower in their SCM (*p* = 0.016) and M (*p* = 0.016). Table 3 shows a summary of biomechanical and viscoelastic properties by muscle and occlusal status.

**TABLE 3:** Biomechanical and viscoelastic properties of the Sternocleidomastoid (SCM), Erector Spinalis Longissimus (ES) and Masseter (M) muscles when grouping subjects according with those showing correct occlusion (Correct) and those with presence of one or more malocclusal traits (Malocclusion).

	SCM			ES			M			*P*_(muscle)_
				
	Correct	Malocclusion	*P*_(group)_	Correct	Malocclusion	*P*_(group)_	Correct	Malocclusion	*P*_(group)_	
Frequency (Hz)	17.1 ± 3.21	13.79 ± 2.7	0.03	14.51 ± 1.53	15.63 ± 1.87	0.023	16.32 ± 2.58	15.85 ± 1.52	0.022	<0.001
Stiffness (N/m)	330.4 ± 117.9	220.5 ± 73	0.021	261.3 ± 73.3	317.8 ± 92.2	0.019	333.5 ± 80.7	324.2 ± 60.2	0.020	<0.001
Decrement	1.08 ± 0.24	1.04 ± 0.1	0.487	0.89 ± 0.19	0.97 ± 0.15	0.125	1.44 ± 0.28	1.75 ± 0.14	0.015	<0.001
Relaxation (ms)	16.33 ± 4.88	22.95 ± 5.01	0.016	17.78 ± 2.48	16.39 ± 3.39	0.012	16.34 ± 3.38	17.21 ± 2.76	0.016	<0.001

All the analyzed muscle variables (oscillation frequency, dynamic stiffness, elasticity, and relaxation time) were statistically different among muscles (*p* < 0.001). The overall interaction between each muscle variable and the severity of dental malocclusion was statistically significant (oscillation frequency, *p* = 0.031; dynamic stiffness, *p* = 0.04; elasticity, *p* = 0.001; and relaxation time, *p* = 0.037).

**FIGURE 4:**
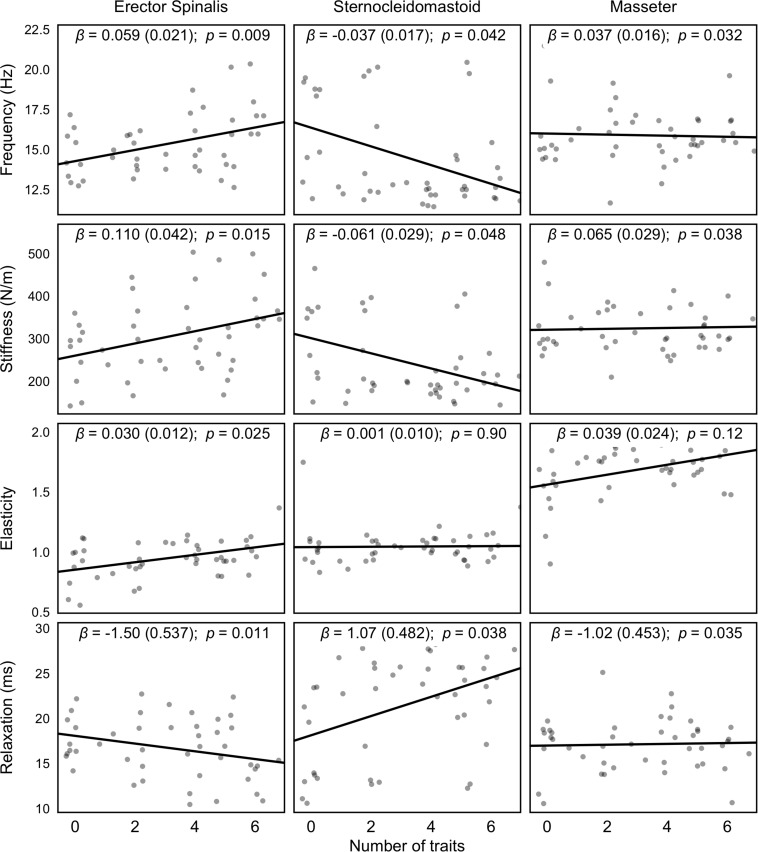
Regression lines of each muscle variable analyzed with MyotonPRO^®^ according to the severity of malocclusion (0 indicates correct occlusion; the higher number of malocclusal trait, the most severity of dental malocclusion). β represents the Estimate Interaction coefficient.

Oscillation frequency had a negative slope for the severity of dental malocclusion in the SCM (*p* = 0.042), but on the contrary, positive associations for ES (*p* = 0.009) and M (*p* = 0.032). Similar results were found for dynamic stiffness, with a negative slope for SCM (*p* = 0.048) and positive for ES (*p* = 0.015) and M (*p* = 0.038). On the contrary, relaxation time had a positive slope for SCM (*p* = 0.038), but negative slopes were observed for ES (*p* = 0.011) and M (*p* = 0.035). Elasticity showed a similar tendency among muscles, with no statistically significant differences (*p* = 0.062) (Figure 4).

A detailed analysis of the influence of malocclusal traits on the muscle properties reveals that angle class, orthodontic treatment, crowding, midline deviation, anterior open bite, overbite, overjet, and version influenced significantly on the frequency, stiffness, elasticity, and relaxation time of some of the muscles analyzed (Table 4).

**TABLE 4:** Biomechanical and viscoelastic properties of the Sternocleidomastoid (SCM), Erector Spinalis (ES) and Masseter (M) muscles influenced by malocclusal traits.

		Frequency		Stiffness		Elasticity		Relaxation	
					
Trait	Muscle	*ES*	*p _(ES)_*	*ES*	*p _(ES)_*	*ES*	*p _(ES)_*	*ES*	*p _(ES)_*
Angle III	ES	0.69	0.012	0.69	0.028	0.42	0.24	–0.41	0.072
	SCM	–0.81	0.083	–0.88	0.10	0	1.00	0.85	0.056
	M	–0.02	0.96	0.07	0.82	1.46	0.016	0.07	0.81
Orthodontics	ES	–0.59	0.089	–0.47	0.17	–0.62	0.070	0.22	0.51
	SCM	0.81	0.019	0.65	0.056	–0.32	0.34	–0.75	0.031
	M	0.07	0.85	0.62	0.070	–0.17	0.61	–0.5	0.14
Crowding	ES	0.59	0.062	0.41	0.18	0.5	0.11	–0.39	0.21
	SCM	–0.28	0.36	–0.35	0.26	–0.1	0.75	0.19	0.54
	M	0.15	0.62	0.22	0.47	0.62	0.049	–0.18	0.55
Midline deviation	ES	0.32	0.27	0.54	0.068	0.86	0.005	–0.37	0.21
	SCM	–1.44	<0.001	–1.26	<0.001	0.14	0.64	1.56	<0.001
	M	–0.32	0.28	–0.31	0.30	0.4	0.18	0.37	0.21
Diastema	ES	–0.37	0.43	–0.28	0.54	1	0.059	0.29	0.52
	SCM	–0.02	0.97	0.09	0.84	0.14	0.75	0.11	0.81
	M	–0.35	0.43	–0.09	0.84	0.78	0.099	0.12	0.80
Missing teeth	ES	–0.3	0.60	–0.37	0.51	0.84	0.19	0.29	0.61
	SCM	0.39	0.50	0.48	0.41	0.09	0.87	–0.33	0.57
	M	–0.22	0.70	0.45	0.43	0.91	0.14	–0.36	0.52
Anterior open bite	ES	2.2	<0.001	1.95	<0.001	0.52	0.21	–1.77	<0.001
	SCM	–1.06	0.011	–0.94	0.025	–0.02	0.96	0.78	0.055
	M	–0.11	0.77	0.19	0.63	0.71	0.079	–0.02	0.95
Overbite	ES	0.46	0.12	0.43	0.15	0.26	0.38	–0.39	0.19
	SCM	–0.17	0.57	–0.09	0.76	–0.04	0.89	0.23	0.43
	M	0.43	0.15	0.28	0.34	0.72	0.017	–0.27	0.36
Overjet	ES	0.88	0.005	0.54	0.076	0.55	0.073	–0.33	0.28
	SCM	–1.25	<0.001	–1.08	0.001	0.22	0.47	1.26	<0.001
	M	–0.33	0.27	–0.3	0.32	0.63	0.046	0.5	0.099
Version	ES	0.63	0.093	0.59	0.11	0.31	0.39	–0.34	0.35
	SCM	–1.05	0.005	–1.07	0.005	–0.3	0.41	0.75	0.042
	M	0.23	0.53	0.16	0.65	0.5	0.18	–0.1	0.79

## Discussion

The present study attempted to determine (i) the dynamic stability measured with the MSEBT under opposing dental occlusion conditions (CR/ICP) and (ii) the biomechanical and viscoelastic properties of the masticatory (masseter), cervical (SCM), and postural muscles (erector spinae) measured with MyotonPRO^®^ in subjects with malocclusal traits and ideal dental occlusion. The main findings were the following: (i) the SEBT composite score was significantly better when dental occlusion was set in CR (*p* < 0.001); (ii) none of the malocclusal traits by themselves influenced the stability index measured with the SEBT. However, the SEBT composite score was significantly higher in subjects with correct occlusion in comparison with those showing one or more malocclusal traits (*p* = 0.04). (iii) The biomechanical properties of the analyzed muscles were influenced by the presence of malocclusal traits.

Our results indicated that dental occlusion differentially contributed to the dynamic stability, with an improvement when dental occlusion was set in CR disregarding the dominant and non-dominant lower limb (*p* < 0.001) (Figure 2). These findings are fairly consistent with the research of Heit et al. (2015) who observed a significant improvement (*p* < 0.001) in balance measured with the MSEBT when the mandible of the subjects were placed in a physiological rest position comparing with measurements made in ICP. The improvement in the SEBT score in the CR position supports the hypothesis of a relationship between balance ability and the stomatognathic system. Previously, the influence of dental occlusion on the body balance in unstable but not in stable conditions has been addressed by several researchers as well as our study group (Tardieu et al., 2009; Julià-Sánchez et al., 2015, 2016). Therefore, the influence of dental occlusion on balance seems to be related to a reorganization of the sensory information for balance control. In this regard, indications for a different contribution of the sensory information for the balance control have been provided when changing the firm support base to an unstable one (Peterka, 2002). This finding offers more clarity to the lack of scientific agreement in the relationship between dental occlusion and body balance, which might be strongly influenced by the external perturbations (in the present research, dynamic balance) as reported in a recent review (Julià-Sánchez et al., 2019). In the majority of sports, subjects are exposed to external forces that promote a constant need of postural control regulation. The maintenance of the head posture is essential for proper balance control, and it is gained through the interaction between craniocervical bones, myofascial structures and dental occlusion (Michelotti et al., 2011). In this regard, the use of occlusal splints to improve the physical performance has been reported in several sports like basketball, showing an improvement in the quadriceps force (Baldini et al., 2012), and in competitive athletes of boxing, swimming, and rugby (D’Ermes et al., 2012), demonstrating better distribution of muscle loads and less muscular effort. In this regard, occlusal splints were suggested to elicit better control of neuromotor coordination, resulting in improved efficiency and effectiveness of specific motor tasks (D’Ermes et al., 2012).

In our research, any of the analyzed malocclusal traits negatively influenced balance ability assessed with the MSEBT. Thus, the statistically significant differences in the SEBT score observed for all traits when comparing balance in CR vs. ICP (*p* < 0.001 for all characters except for no version, *p* = 0.007) can be attributed to the change in the mandible position, as explained above, and not for presenting any specific maloccusal trait. However, results of the present study must be interpreted with some caution because of the small sample size of subjects with each of these occlusal traits, and more studies should be done to better clarify the true relationship between posture and dental occlusion characteristics. A remarkable finding of the present study was that SEBT composite score was significantly higher in subjects with correct occlusion (without any malocclusal trait) than those with malocclusion (showing one or more malocclusal traits) (*p* = 0.04) regardless of the dental occlusion condition measured (CR/ICP) (Figure 3). In addition, both groups improved the SEBT composite score when placing the mandible position from ICP to CR (malocclusion, *p* < 0.001; correct occlusion, *p* = 0.019). An interesting finding in our study was that subjects with correct occlusion showed better balance than subjects with malocclusion in the measurements of SEBT made in ICP (*p* = 0.047), whereas when correcting the mandible position (CR), the balance of the group with correct occlusion and the group with malocclusion was similar (*p* = 0.12). A plausible explanation for that finding is that the cotton rolls placed between the two arches distributed the occlusal load onto several teeth, reducing the precision of proprioceptive periodontal information (Ferrario et al., 1996). Thus, minimizing the impact of incongruous occlusal contacts could explain the similar stability achieved in the CR mandibular position disregarding the presence of malocclusal traits. This finding suggests a strong influence of dental occlusal status on the balance stability, with subjects showing some malocclusal traits benefiting more than those with an correct occlusion when placing the mandible in a position independent of dental occlusion. In accordance, Milani et al. (2000) reported that wearing oral splints long time causes fluctuations on dynamic balance, thus confirming the effects upon the postural control in subjects with dental malocclusion.

Regarding the muscle properties analysis, we found that the oscillation frequency, dynamic stiffness, elasticity, and relaxation time were statistically significant different between muscles (*p* < 0.001). These results seem logical when considering that muscle tone depends on several factors such as stretch reflex, vestibular and tonic neck reflexes, crossed extensor reflex, body scheme, fatigue level, and torque performed during movement (Pérennou, 2012; Ivanenko et al., 2013; Gogola et al., 2014). Postural stability can be maintained in balanced equilibrium by passive resting muscular tone. These passive viscoelastic muscle properties are independent from CNS activation and electromyographic activity and are conditioned by mechanical myofascial tissue properties (Mense and Masi, 2010; Nair et al., 2016). The MyotonPro is a non-invasive device that allows to explain variations in mechanical properties from resting muscular tone. Many studies described a solid relationship between altered muscle mechanical properties quantified using MyotonPro and some injuries as low back pain (Nair et al., 2016) and ankylosing spondylitis (Andonian et al., 2015). On the other hand, CNS-stimulated muscle tone depends on active contractions and can be detected by electromyography devices (Masi et al., 2010). An increase in this happens because CNS-activated tension is greater than the resting tone (Masi and Hannon, 2008). A change in the mechanical properties, recorded by MyotonPro from different body tissues, has been described as an important intrinsic risk factor for injury and overtraining (Gabbe et al., 2004; Gavronski et al., 2007). These properties could be explained through the change in four different variables: frequency (Hz), stiffness (N/m), elasticity (log decrement), and relaxation time (ms) (Orner et al., 2018). Interestingly, the interaction of each variable with the severity of dental malocclusion showed a positive slope according to the number of malocclusal traits (oscillation frequency, *p* = 0.031; dynamic stiffness, *p* = 0.04; elasticity, *p* = 0.001; and relaxation time, *p* = 0.037). From earlier investigations, it is known that the number of occlusal contacts and masticatory muscular function are associated with superior muscle activity when dental occlusion is more harmonious (Ferrario et al., 2002). These results are in line with previous research reporting a significant reduction in the rest activity of postural muscles (SCM, ES, and soleus) in subjects affected by dental malocclusions after a neuromuscular balance of the occlusion to achieve an optimum occlusal relationship (Bergamini et al., 2008). This may well be related to the influence of trigeminal afferences from dental occlusion which interact with the muscle system at different levels. Experimental evidence of trigeminal influences on the neck and cervical muscles activity (Alstermark et al., 1992) as well as in postural control (Delfini et al., 2000) has been previously reported.

As expected, the slope in oscillation frequency and dynamic stiffness, according to the severity of dental malocclusion, showed a positive trend for ES (*p* = 0.009; *p* = 0.015) and M (*p* = 0.032; *p* = 0.038). Interpretation criteria means how higher values of stiffness and elasticity seem to indicate greater levels of tension, while lower relaxation time respond to a smaller dissipation of mechanical energy (Gavronski et al., 2007). Thus, the presence of malocclusal traits increase the level of tension in the ES and M muscles. It was previously shown that as the quality and strength of the muscle fibers decrease, the stiffness of the muscle increases (Akagi et al., 2015; Eby et al., 2015). Therefore, the presence of severe dental malocclusion could lead to a muscle overuse that may cause an impairment in the capillary blood flow (Bron and Dommerholt, 2012). On the contrary, the slope of SCM showed a negative trend in oscillation frequency (*p* = 0.042) and dynamic stiffness (*p* = 0.048), which means that a loss of tension appears as the number of malocclusal traits increases. This may be explained by the fact that the SCM differs from the ES, as it is a dynamic postural muscle and not a static one as the ES. Thus, it is likely that the stiffness of other cervical postural muscles could be increased. However, more studies should focus on the analysis of the muscle activity on the back muscles to better elucidate these findings. Predictably, the relaxation time had a negative slope for ES (*p* = 0.011) and M (*p* = 0.035) but positive for SCM (*p* = 0.038). This is explained because stiffness and elasticity increase while relaxation decreases and could be interpreted as an increase in tension levels due to a greater demand of the nervous system to counteract gravity action (Schneider et al., 2015). Finally, no statistically significant differences were observed for elasticity, which showed a similar tendency among muscles (*p* = 0.062).

The functional coupling between dental occlusion and neck muscles activity had been described in the past years (Ferrario et al., 2003). A remarkable finding of the present study was that angle class, orthodontic treatment, crowding, midline deviation, anterior open bite, overbite, overjet, and version influenced significantly at least one of the muscle properties analyzed (see Table 4). Direct comparison of the results from the present study with previous research is limited because none of the previous researchers analyzed the influence of dental occlusion status on skeletal muscle activity. However, associations between some of these specific malocclusal traits and muscle activity have been reported. In this regard, anterior open bite was correlated to a weak elevator muscles activity (Bakke and Michler, 1991) and also to alterations in the chewing pattern of the T and M muscles, most probably because the lack of inputs from the anterior guidance causes an alteration of the motor scheme (Piancino et al., 2012). As opposed to those findings, we did not see any influence of open bite on the masseter muscle activity; however, we measured rest activity in contrast with those previously mentioned. In addition, angle class II occlusion has been correlated to alterations in the muscle activity pattern of masticatory muscles (M and T) (Gadotti et al., 2005). We did not find differences between angle class and masseter properties, but there were statistically significant differences in the ES and SCM properties. In addition, it is important to note that some of these malocclusal traits had been previously shown to cause impairments in the postural control on unstable platform (Julià-Sánchez et al., 2015). Thus, we hypothesize that the altered muscular pattern observed in the postural muscles, specifically the increase in stiffness reported in the ES, according to the severity of dental malocclusion may play a role in the impairment of postural control. Interestingly, dental malocclusion influenced more on the muscle properties of postural muscles than those of masticatory muscles. A plausible explanation for this finding could be the fact that measurements were made with subjects in supine position to evaluate the influence of dental occlusion on posture. Thus, masseter was measured at rest, while SCM and ES were measured when contributing to maintain posture in an upright position.

The following limitations of our study have to be mentioned: the small numbers within subgroups of different malocclusal traits make it difficult to determine a true relationship between posture and dental occlusion characteristics; the analysis of muscle activity of some masticatory (e.g., temporalis) and postural (e.g., occipital, splenium, and trapezoidal) muscles would add more information on the influence of dental occlusion on muscle chains involved in posture and the interconnections between masticatory and postural muscles. Otherwise, the findings reported here shed new light on the influence of dental occlusion on the masticatory and postural muscle properties and contributes to the growing body of research that reinforces the influence of dental occlusion on dynamic balance, as suggested by the authors in previous research. This study has, for the first time, raised important questions about the effect of not only the dental occlusion status but also the influence of each malocclusal trait on the muscle properties. Future research should focus on determining the influence of dental malocclusion on postural muscles in a relaxed position and also on evaluating the effects of changing the mandible position among the muscle properties of postural muscles.

## Conclusion

Although without irrefutable evidences of a non-specific effect, dental occlusion likely conditioned dynamic balance. In addition, dental occlusion seems differently to influence the biomechanical and viscoelastic properties depending on the muscle group analyzed and the presence of specific malocclusal traits.

These findings will provide a basis for further studies examining skeletal muscle activity according to dental occlusion status.

## Data Availability Statement

The datasets generated for this study are available on request to the corresponding author.

## Ethics Statement

The studies involving human participants were reviewed and approved by the Universitat de Lleida (UdL) Ethics Committee. The patients/participants provided their written informed consent to participate in this study.

## Author Contributions

All authors read and concurred with the content in the final manuscript. SJ-S, JÁ-H, FC, and RC-S participated in the acquisition and interpretation of the data. RC-S performed the statistical analysis. SJ-S, JÁ-H, and MB participated in the drafting and critical revision of the manuscript.

## Conflict of Interest

The authors declare that the research was conducted in the absence of any commercial or financial relationships that could be construed as a potential conflict of interest.
